# Deep-Blue and Narrowband-Emitting Carbon Dots from a Sustainable Precursor for Random Lasing

**DOI:** 10.1021/acsanm.4c06734

**Published:** 2025-01-30

**Authors:** Junkai Ren, Jiong Liu, Bing Wei, Wenfei Zhang, Ludvig Edman, Jia Wang

**Affiliations:** a The Organic Photonics and Electronics Group, Department of Physics, Umeå University, Umeå SE-90187, Sweden; b Key Laboratory of Optoelectronic Devices and Systems of Ministry of Education and Guangdong Province, College of Physics and Optoelectronic Engineering, Shenzhen University, Shenzhen 518060, China; c School of Physics, Xidian University, Xi’an 710071, China; d Wallenberg Initiative Materials Science for Sustainability, Department of Physics, Umeå University, Umeå SE-90187, Sweden

**Keywords:** carbon dots, deep-blue emission, narrowband emission, high photoluminescence quantum yield, sustainable precursor, random lasing

## Abstract

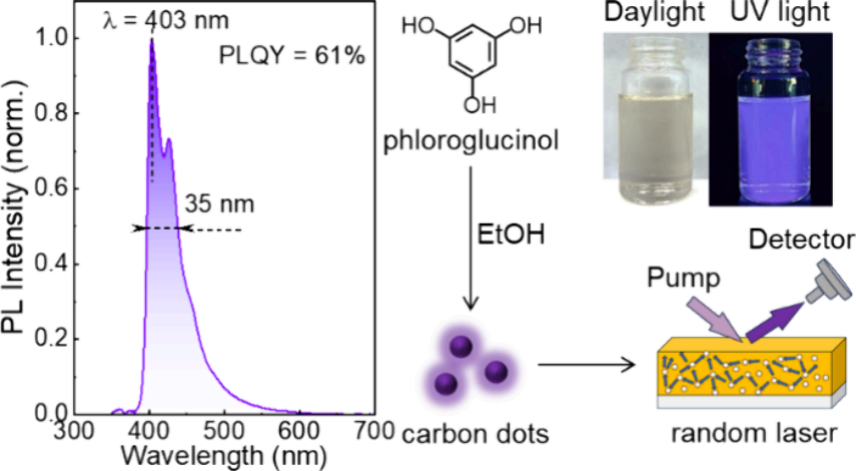

Deep-blue (DB) emitters that feature high photoluminescence quantum yield (PLQY) and narrow spectral bandwidth are desired for a variety of optoelectronic applications, particularly for lighting, illumination, and lasing. Currently favored DB emitters constitute quantum dots comprising cadmium or lead and organic compounds derived from petroleum, but they suffer from toxicity and sustainability issues. Here, we report the solvothermal synthesis of DB-emitting carbon dots (**DB-CD**s) using bioderivable phloroglucinol as the sole starting material, which exhibit a peak emission wavelength of 403 nm, narrow spectral full width at half-maximum of 35 nm, and high PLQY of 61% in ethanol. The **DB-CD**s with a planar structure are demonstrated to comprise distinct graphene segments in a polyether-cross-link network, with the former functioning as the fluorophore. The application merit of the **DB-CD**s is exemplified by their implementation as the gain medium in a random laser device, which exhibits a threshold optical power density of 40.5 kW cm^–2^. This study thus demonstrates a path toward efficient and sustainable deep-blue emitters, which can be exploited in practical applications.

## 1 Introduction

High-efficiency and narrowband deep-blue emitters are a fundamental component in a wide range of optoelectronic applications, including solid-state lighting, high-density information storage, and high-quality color displays.^1,2^ This critical enabling capacity has spurred the development and utilization of a wide variety of different deep-blue emitting materials. For instance, colloidal quantum dots (QDs) are commonly employed deep-blue emitters because of their high photoluminescence quantum yield (PLQY), narrow PL spectrum, high stability, and opportunity for low-cost solution processing.^3−8^ However, QDs typically rely on toxic metals, such as Cd^3,4^ or Pb,^5,6^ and/or critical raw materials (CRMs),^7,8^ which is concomitant with serious environmental concerns. The development of metal-free deep-blue emitters, in the form of small molecules^9,10^ and polymers,^11,12^ then represents an improvement, but their synthesis is typically complex, costly, and dependent on petroleum-derived and therefore nonsustainable chemicals.

Emissive carbon dots (CDs) can in contrast be completely devoid of metals and CRMs and be synthesized by facile and cost-efficient methods using solely biobased starting materials.^13,14^ For instance, Yang et al. synthesized red-emitting CDs from taxus-leaf biomass, with a PL peak of 673 nm and notably narrow full width at half-maximum (fwhm) of the PL spectrum of 20 nm, for application in bioimaging;^15^ our group reported on the synthesis of red-emitting CDs using birch-leaf biomass as the starting material for application in electroluminescent devices;^16^ Fan and colleagues synthesized sky-blue to orange emitting CDs from bioderivable phloroglucinol precursors^17^ whereas Zbořil et al. realized UV-emitting CDs from bioderivable folic acid, which exhibited a PL peak of 390 nm, PLQY of 55%, and fwhm of 60 nm.^18^

However, the corresponding reports on deep-blue and narrowband emitting CDs are unfortunately few.^19−23^ We note that Sargent et al. reported on the chemical conversion of 1,5-diaminonaphthalene and citric acid into CDs, which exhibited a PL peak of 433 nm, PLQY of 70%, and spectral fwhm of 35 nm,^19^ and that Tong and colleagues synthesized CDs from *o*-phenylenediamine and malic acid, featuring a PL peak of 415 nm, PLQY of 48%, and fwhm of 60 nm.^21^ However, although the deep-blue PL performance of these previous CDs indeed is promising, we find that their synthesis invariably relies on the use of at least one petroleum-derived precursor, which is a drawback from a sustainability viewpoint.

Here, we address this issue through our report on the synthesis of deep-blue emitting CDs (DB-CDs) by solvothermal conversion of a single bioderivable phloroglucinol precursor in ethanol. The **DB-CD**s exhibit efficient deep-blue and narrowband PL in ethanol solution, characterized by a peak wavelength of 403 nm, high PLQY of 61%, and fwhm of 35 nm. Structural and optical characterization indicates that the **DB-CD**s exhibit a planar nanostructure, comprising distinct graphene segments dispersed in a polyether-cross-link network, with the former functioning as the distinct fluorophore. Finally, the practical potential of these **DB-CD**s is showcased by employing them as the gain medium in a random laser device, featuring a threshold optical power density of 40.5 kW cm^–2^. These results demonstrate that **DB-CDs** are well-suited for development in random lasing applications, offering significant sustainability benefits.

## 2 Experimental Section

### 2.1 Synthesis

The **DB-CD**s were synthesized by the solvothermal conversion of phloroglucinol (Sigma-Aldrich, Germany, ≥ 99.0%). 0.5 g (4 mmol) of phloroglucinol was dissolved in 10 mL of absolute ethanol (VWR, ≥ 99%) and formed a light-yellow solution. The solvothermal reaction was carried in an autoclave reactor at 200 °C for 24 h. The crude product was purified by column chromatography, with silica gel (pore size = 60 Å, bead size = 35–70 μm, Fisher Scientific) being the stationary phase and a mixture of dichloromethane (HPLC, Fisher Scientific) and methanol (HPLC, Fisher Scientific) as the eluent. The volume ratio of the dichloromethane:methanol eluent was gradually changed from 20:1 to 10:1 during the purification procedure. A colorless fraction was first collected followed by a deep-blue luminescent fraction and finally a cyan luminescent fraction while a black nonluminescent fraction remained at the top of the column. The deep-blue luminescent fraction was collected and dried by rotary evaporation at 30 °C. The collected solid-state **DB-CD**s were redissolved in ethanol for further characterization.

### 2.2 Characterization

To prepare the high-resolution transmission electron microscopy (HRTEM) sample, a **DB-CD**-in-ethanol solution (50 mg L^–1^) was drop-cast onto an ultrathin carbon-coated copper grid. The HRTEM images were recorded with a cryogenic microscope (Titan Krios, Thermo Fisher Scientific) at an acceleration voltage of 300 kV. A direct electron detector (Falcon 4i) and a postcolumn imaging filter (Selectris) were used for HRTEM for enhancing contrast during imaging.

The samples for X-ray diffraction (XRD) and X-ray photoelectron spectroscopy (XPS) measurements were prepared from a **DB-CD**-in-ethanol solution (5 g L^–1^) by drop-casting onto either a glass substrate or a silicon wafer. The films were ∼100 μm thick after completely drying. The XRD pattern was recorded with an X-ray diffractometer (X’Pert^3^ powder, PANalytical), using a Cu Kα radiation source (λ = 1.54056 Å) operating in a continuous scan mode. The XRD baseline was estimated and removed with the aid of HighScore Plus Software. The XPS was collected with an electron spectrometer (Kratos Analytical, AXIS Ultra DLD), using a monochromatic Al Kα source (*h*ν = 1486.6 eV).

The samples for the diffuse reflectance infrared Fourier transform spectrum (DRIFTS) were prepared by dispersing dried **DB-CD**s in IR-grade KBr (≥99%, Sigma-Aldrich). The data were collected by a vacuum-bench spectrometer (IFS 66/S, Bruker) operating in diffuse reflectance mode. The baseline of the DRIFTS spectrum was estimated and removed by using OPUS 5.0 software. For the Raman spectrum, a Qontor spectrometer (Renishaw) with a continuous solid-state laser source (wavelength of 405 nm) was used. The luminescent background of the **DB-CD**s in the Raman spectrum was estimated and subtracted using WiRE 2.0 Software. The nuclear magnetic resonance (NMR) spectra of **DB-CD**s were recorded using an Avance II 400 MHz instrument (Bruker, Germany) by dissolving them in deuterated reagents.

**DB-CD**s were analyzed by a 6230 time-of-flight mass spectrometer TOF-MS (Agilent) equipped with the Agilent 1290 Infinity II UPLC. A 3 μL portion of **DB-CD**s in methanol (8 g L^–1^) was injected onto one C18 column (Extend-C18, 1.8 μm, 2.1 × 50 mm, Agilent Technologies). Then, they were separated using the gradient of increasing mobile phase B as follows. Flow rate is 0.4 mL min^–1^. Mobile phase A is 0.1% formic acid (HPLC grade, Merck) in H_2_O, and mobile phase B is 0.1% formic acid in acetonitrile (HPLC grade, Merck). The gradient starts with 20% B, rises to 100% B over 7 min, and then holds at 100% B for 2 min. Then, it drops to 20% B in 0.1 min and then holds for 3 min. The total length is 12 min. The eluent was sprayed into the TOF through Dual AJS electrospray ionization (ESI) in positive mode. Dual AJS ESI settings are as follows: drying gas 10 L min^–1^, nebulizer 40 psig, sheath gas temperature is 350 °C, sheath gas flow 12 L min^–1^, VCap 4000 V and nozzle voltage is 2000 V. For TOF-MS analysis, the fragmentor was 250 V, skimmer 65 V and Oct1 RF Vpp 750 V. The mass range is set to between 100 and 3200 *mz*^–1^ with acquisition rate of 1.5 spectra s^–1^. Data analysis was done by an Agilent MassHunter Qualitative Analysis (version B.07.00).

The UV–Vis absorption spectra were recorded with a two-beam scanning spectrophotometer (Lambda 1050, PerkinElmer). The PL spectra were collected with a fluorescence spectrometer (FLS1000, Edinburgh) using a 450 W ozone-free xenon arc lamp as the excitation source. The PLQY was measured with a spectrometer (C9920–02G, Hamamatsu) equipped with an integrating sphere. For the stress test, UV illumination was delivered by a UV lamp (ZF-20D, λ_peak_ = 365 nm, power intensity = 230 W m^–2^). The transient PL decays were measured with a standard photomultiplier (PMT-900, Edinburgh) as the detector, using a pulsed diode laser (λ_exc_ = 375 nm and pulse width = 60 ps) as the excitation source. The fluorescent lifetime was calculated by fitting the transient data with the Fluoracle Software.

The **DB-CD**s were dispersed in ethanol at 6.25 g L^–1^ and the SiO_2_ particles (diameter size = 0.87 μm) were dispersed in epoxy resin at 40 g L^–1^. The three components were blended together in a **DB-CD**:SiO_2_:epoxy mass ratio of 1:110:850. The blend solution was spin-coated onto a quartz substrate (area = 1 × 1 cm^2^) at 450 rpm for 15 s and then baked at 60 °C for 120 min. The dry film thickness of the spin-coated blend film was 700 μm. The excitation beam, which was generated by a neodymium-doped yttrium aluminum garnet (Nd:YAG) pulsed laser (6 ns, 10 Hz, Continuum Surelite, California, USA) and an optical parameter oscillator (Continuum Horizon, California, USA), was focused onto a small spot (diameter: 1000 μm) on the **DB-CD**s film, whereas the resulting emission was collected by a spectrophotometer (HORIBA iHR320. Minami-ku, Kyoto, Japan) equipped with an optical fiber.

## 3 Results and Discussion

### 3.1 Synthesis and Optical Characterization

**Figure 1:**
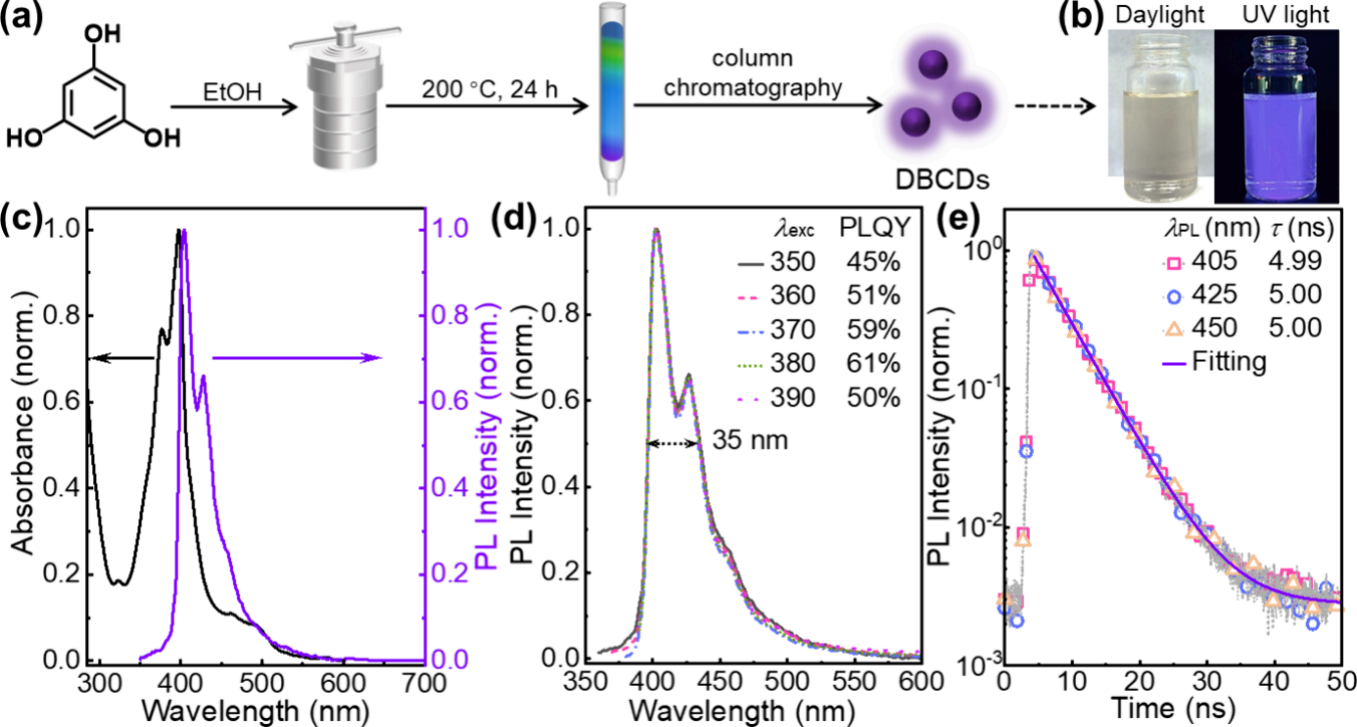
(a) Schematic illustrating the key steps in the solvothermal conversion of the phloroglucinol precursor in ethanol to **DB-CD**s, and the subsequent purification by column chromatography. (b) Photographs of the **DB-CD**s in ethanol (solute concentration = 50 mg L^–1^) under daylight illumination (left) and UV illumination (right, λ_exc_= 365 nm). (c) The normalized UV–vis absorption spectrum (left *y* axis) and the normalized PL spectrum (right *y* axis) of a **DB-CD** in ethanol solution with a solute concentration of 10 mg L^–1^. (d) Normalized PL spectrum as a function of the excitation wavelength, as identified in the inset. The corresponding PLQY values are displayed in the same inset. (e) The normalized transient PL decays as a function of the recorded emission wavelength. The experimental data are shown as open symbols, while the solid line represents the monoexponential fit with a constant background. The inset displays the emission lifetime τ value extracted from the fitting.

Figure 1(a) is a schematic presenting the key steps in the solvothermal chemical conversion of the phloroglucinol starting material into **DB-CD**s as reported elsewhere.^24^ The resulting crude solution was purified by using silica-gel column chromatography. During the process, a colorless fraction emerged first followed by a deep-blue luminescent fraction and then a cyan luminescent fraction, while a nonemissive insoluble remained at the top of the column. The deep-blue luminescent fraction was collected and dried to obtain solid-state **DB-CD**s, which subsequently were redissolved in ethanol for further characterization.

Phloroglucinol is considered a desired precursor for producing narrow-bandwidth CDs due to its triangle-symmetric molecular structure. This structural feature enables a trimolecular reaction pathway during the cyclization process, which involves the adjacent active −OH and −H groups.^17^ Additionally, phloroglucinol is a bioderived precursor that can be directly isolated from various plants, including roses and acacia, or alternatively synthesized from a range of biomass sources, such as fruit trees and certain bacteria.^24^ Ethanol, utilized in our synthesis, is also a bioderived green solvent. It is important to note that the entire synthesis process for **DB-CD**s is conducted under ambient air conditions and does not involve hazardous chemicals. As a result, the synthesis of **DB-CD**s is considered to be sustainable.

Figure 1(b) presents two photographs of **DB-CD** in ethanol solution (solute concentration = 50 mg L^–1^), which exhibits a light-yellow appearance under daylight illumination (left photograph) and vibrant deep-blue emission during exposure to UV light (right photograph). Figure 1(c) presents the normalized UV–vis absorption spectrum (left *y* axis) and the normalized PL spectrum (right *y* axis) of dilute **DB-CD** in ethanol solution (10 mg L^–1^). The primary absorption peak appears at 396 nm, accompanied by a prominent higher-energy shoulder at 375 nm, and the fwhm of the major absorption envelope is 40 nm. The major PL peak is centered at 403 nm, with two lower-energy shoulder peaks distinguishable at 426 and 457 nm and the fwhm of the PL envelope being 35 nm.

Importantly, the PL measurement verifies that the synthesized **DB-CD**s indeed exhibit the desired deep-blue and narrowband emission. More specifically, Figure S1 shows that the color coordinates of the **DB-CD**s in the Commission Internationale de l’Eclairage (CIE) 1931 diagram are (0.165, 0.054), which is close to the blue chromaticity standard of (0.131, 0.046), as defined by the Rec. 2020.^25^ Moreover, another common criterion of a deep-blue emitter is that its CIE *y*-coordinate (CIE_*y*_) should be below 0.1,^26^ which is also fulfilled by the **DB-CD**s with a CIE_*y*_ of 0.054.

We wish to call attention to that both the absorption and the PL spectra in Figure 1(c) are notably narrow and structured, that they are essentially each other’s mirror image, and that the Stokes shift is very minor at 7 nm. These observations imply that the observed shoulder peaks in absorption and emission correspond to vibronic peaks of the same electronic transition and that the optically active part of the **DB-CD** is positioned in a rigid environment and exhibits a similar molecular conformation in the ground state and the first excited (emissive) state.^27,28^Figure 1(d) shows the normalized PL spectra and the corresponding PLQY (see inset) of a dilute **DB-CD** in ethanol solution (10 mg L^–1^) as a function of excitation wavelength, as detailed in the inset. Importantly, the PLQY is high, as desired, with the peak value of 61% measured with an excitation wavelength of 380 nm (see Figure S2). The PL spectral shape is observed to be highly independent of the excitation wavelength, which suggests that the emission originates from a single type of luminophore and the intrinsic states of **DB-CD**s.^29,30^

Figure 1(e) presents the normalized transient PL decays of the **DB-CD** in ethanol solution at three different PL detection wavelengths (see the inset). The selected emission wavelengths correspond to the PL main peak, the first vibronic peak, and the second vibronic peak of the **DB-CDs**. The transient PL decays for all three PL wavelengths were fitted with high accuracy, using a monoexponential function (along with a constant background), as demonstrated by the good agreement between the solid fitting lines and the measured data. The derived emissive lifetime is 5.0 ns for all three PL wavelengths, and this short lifetime suggests that the **DB-CD**s emit by the process of fluorescence.^31^ Moreover, the combined observations of a monoexponential decay and a single emissive lifetime support our conclusion above that it is solely one type of fluorophore that emits in the **DB-CD**s.

Figure S3(a) displays the influence of the solvent selection on the PL spectrum and the PLQY of a dilute **DB-CD** solution, with the solvent selection ranging from low-polarity acetone (polarity parameter: E_T_^N^ = 0.355) to high-polarity water (E_T_^N^ = 1).^32^ The PLQY is relatively constant (at 50 ± 10%) and the PL spectral shape rather invariant for all four investigated solvents, although the relative intensity of the shoulder peaks decreases somewhat with increasing solvent polarity. These observations imply that the single **DB-CD** fluorophore is interacting very weakly with the surrounding solvent environment, which in turn suggests that the fluorophore is protected from the solvent by being embedded in the **DB-CD** bulk.

The PL is, in contrast, found to be highly sensitive to the **DB-CD** concentration. Figure S3(b) shows that the PLQY of **DB-CD** in ethanol decreases dramatically with an increasing **DB-CD** concentration, from 51% at 5 mg L^–1^ to 9% at 1000 mg L^–1^. In parallel, the relative intensity of the lower-energy PL peaks increases markedly with an increasing **DB-CD** concentration. These observations suggest that the **DB-CD**s are highly sensitive to aggregation caused quenching.

To gain deeper insights into the intrinsic characteristics of the **DB-CD**s, we have recorded the temperature-dependent PL spectra over a range from 85 to 295 K (see Figure S4). As the temperature increases, a minor red shift in the PL peak is observed from 400 to 403 nm, accompanied by an increase in the fwhm values from 28 to 35 nm. This phenomenon aligns with previous reports on narrow-bandwidth CDs.^17,19^ The behavior can be attributed to reduced electron–phonon coupling, resulting from restricted structural vibrations and distortions at lower temperatures.^17^ Based on this observation, we anticipate that the luminophore in **DB-CD**s exhibits a rigid structure with only moderate conformational effects from the surrounding environment.

### 3.2 Structural Characterization

**Figure 2:**
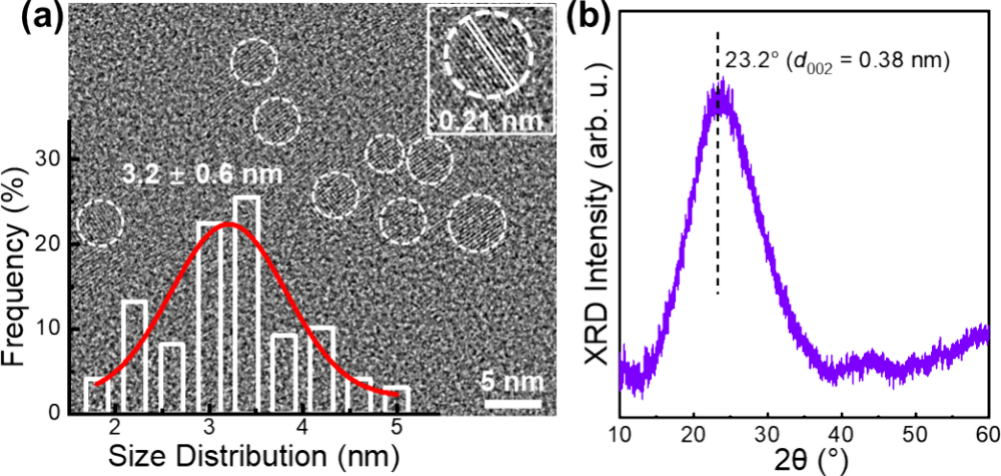
(a) HRTEM image of discrete **DB-CD**s deposited on a carbon film supported by a copper grid. Upper-right inset: enlargement of a single **DB-CD** comprising an ordered lattice structure with an interplanar spacing of ∼0.21 nm. Lower-left inset: a size-distribution histogram of the **DB-CD**s; the red solid line is a fit with a Gaussian function, which yields that the average **DB-CD** diameter is 3.2 nm and that the standard deviation σ is 0.6 nm. (b) XRD pattern of a drop-cast ∼100 μm-thick **DB-CD** film on a glass substrate. The main peak is marked by a dashed line, and the corresponding derived interplanar distance is presented in the parentheses.

Figure 2(a) is an HRTEM image of eight discrete **DB-CD**s on a carbon film on a copper grid support. The dashed circles identify the circular-shaped ordered **DB-CD** structures that can be detected by HRTEM. The inset in the upper right is an enlargement of the HRTEM-detectable structure of a representative single **DB-CD**. We note that the detected “interplanar spacing” of ∼0.21 nm is similar to the characteristic interplanar distance between (100) graphene planes.^33^ We also determined the diameter of this circular-shaped ordered structure for 50 different **DB-CD**s, and the derived diameter distribution is displayed in the histogram in the lower left inset with the red line representing a fit with a Gaussian function. The derived average diameter of the ordered **DB-CD** structure is 3.2 nm, with the standard deviation being 0.6 nm.

Figure 2(b) shows the X-ray diffraction (XRD) pattern of a drop-cast film of **DB-CD**s, approximately 100 μm thick, on a glass substrate. A broad diffraction peak is found at 2θ = 23.2°. With the aid of Bragg’s law, this diffraction peak can be translated to an interplanar distance of 0.38 nm. It is worth mentioning that this spacing is slightly larger than the characteristic spacing of 0.34 nm between the (002) planes of graphite.^34^ Combined with the observations of a graphene-like interior structure of the **DB-CD** (see Figure 2(a)) and the strong aggregation caused quenching (as inferred from Figure S3(b)), we draw the conclusion that the graphene-comprising **DB-CD**s can form graphite-like stacking structures in neat films. The slight increase of the interplanar spacing of the neat **DB-CD** film compared to the interplanar (002) spacing of bulk graphite implies that nongraphene groups are also a significant part of the **DB-CD** structure and that these nongraphene groups are causing an expansion of the **DB-CD** lattice in the *c* direction in neat films.^24,35^

**Figure 3:**
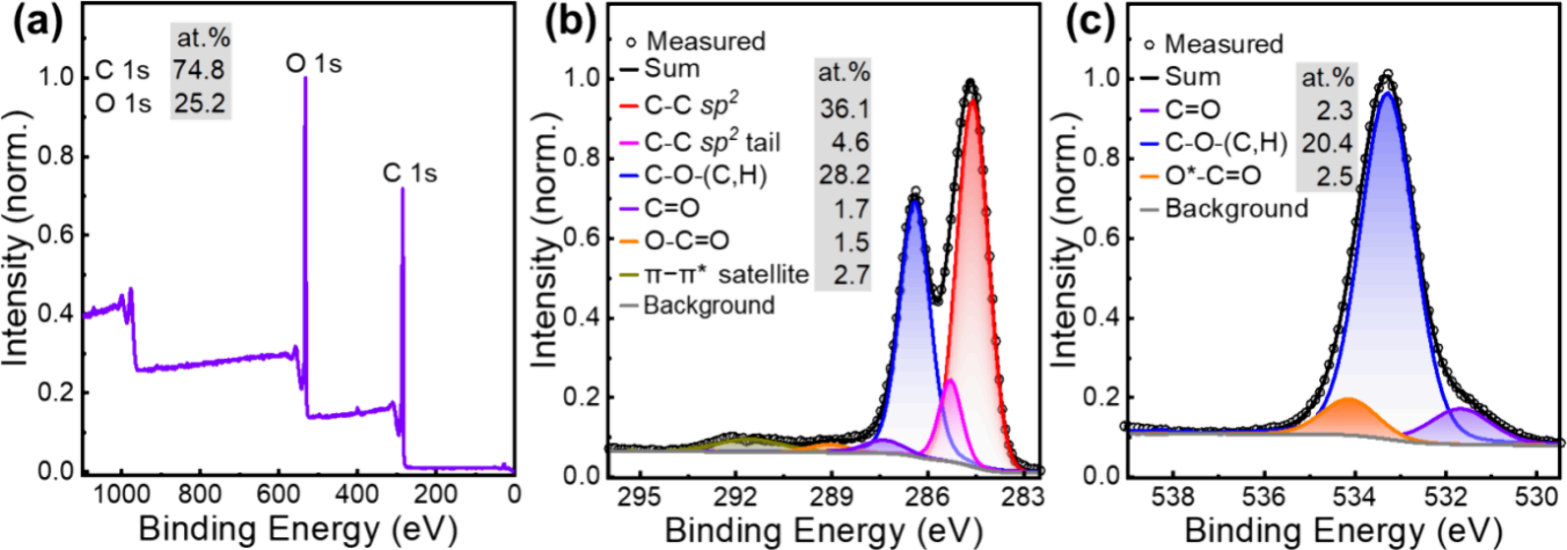
(a) XPS survey spectrum of a drop-cast ∼100 μm-thick **DB-CD** film on a silicon substrate. The inset shows the derived atomic concentrations. (b, c) High-resolution XPS spectra in the (b) C 1s and (c) O 1s regions. The experimental data are depicted by open black circles, while the fitted data are shown as solid colored lines and shaded areas. The solid black lines represent the sum of the fitted data. The inset provides the peak assignments and atomic concentrations of the fitted spectral components.

Figure 3(a) presents an XPS survey spectrum of a drop-cast neat **DB-CD** film. A peak analysis based on the integral area yields that the **DB-CD**s comprise 74.8 at. % carbon and 25.2 at. % oxygen (note that hydrogen is not detected by XPS). Figure 3(b,c) presents high-resolution XPS spectra in the C 1s and O 1s regions, with the fitted XPS peaks indicated by the solid colored lines and colored shadows. The chemical bond assignments and their relative concentrations are displayed in the inset. The observed good agreement between the sum of the fitting spectra (solid black lines) and the measured XPS spectra (open black circles) shows that the analysis is of high accuracy.

The measured C 1s spectrum in Figure 3(b) is deconvoluted into a strong (36.1 at. %) “C–C sp^2^ bond” peak at 284.6 eV, a minor (4.6 at. %) asymmetric “C–C sp^2^ tail” peak at 285.2 eV,^36^ and a minor (2.7 at. %) “π–π* shake-up satellite” at 291.5 eV.^37^ These three “aromatic carbon” peaks summarize to 43.4 at. %, which suggests that roughly half of the atoms (excluding hydrogen) in **DB-CD**s are part of aromatic structures, such as graphene or benzene. We further identify a major (28.2 at. %) C–O–(C,H) peak at 286.4 eV and two minor peaks at 287.4 and 289.0 eV, which we assign to C=O and C=O–O structures, respectively.

The corresponding analysis of the O 1s spectrum in Figure 3(c) results in the identification of a major (20.4 at. %) “C–O–(C,H)” peak at 533.3 eV, a first minor (2.3 at. %) “C=O” peak at 531.3 eV, and a second minor (2.5 at. %) “O*–C=O” peak at 534.1 eV. We call attention to that the observed differences in the atomic concentration of the C–O–C peak in the C 1s and O 1s spectra arise from the structural composition, which includes two carbon atoms (identified in the C 1s spectrum) and one oxygen atom (identified in the O 1s spectrum). Thus, with eqs 1–4 and the data gleaned from the C 1s and O 1s spectra in Figure 3, we can then establish that the “C–O–(C,H)” peak can be separated into a distinct “C–O–C” peak comprising 15.6 at. % C and 7.8 at. % O and a distinct “C–O–H” peak comprising 12.6 at. % C and 12.6 at. % O. Importantly, the observation of a strong “C–O–C” peak suggests that a C–O–C “ether linkage” coupling reaction has taken place during the solvothermal conversion of the phloroglucinol precursor into **DB-CD**s.
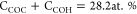
1
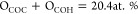
2
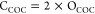
3
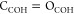
4

Note: C_COC_ and C_COH_ are the total carbon fraction in the C–O–C and C–O–H bonds, respectively, in the C 1s spectrum, while O_COC_ and O_COH_ is the total oxygen fraction in the C–O–C and C–O–H bonds, respectively, in the O 1s spectrum.

**Figure 4:**
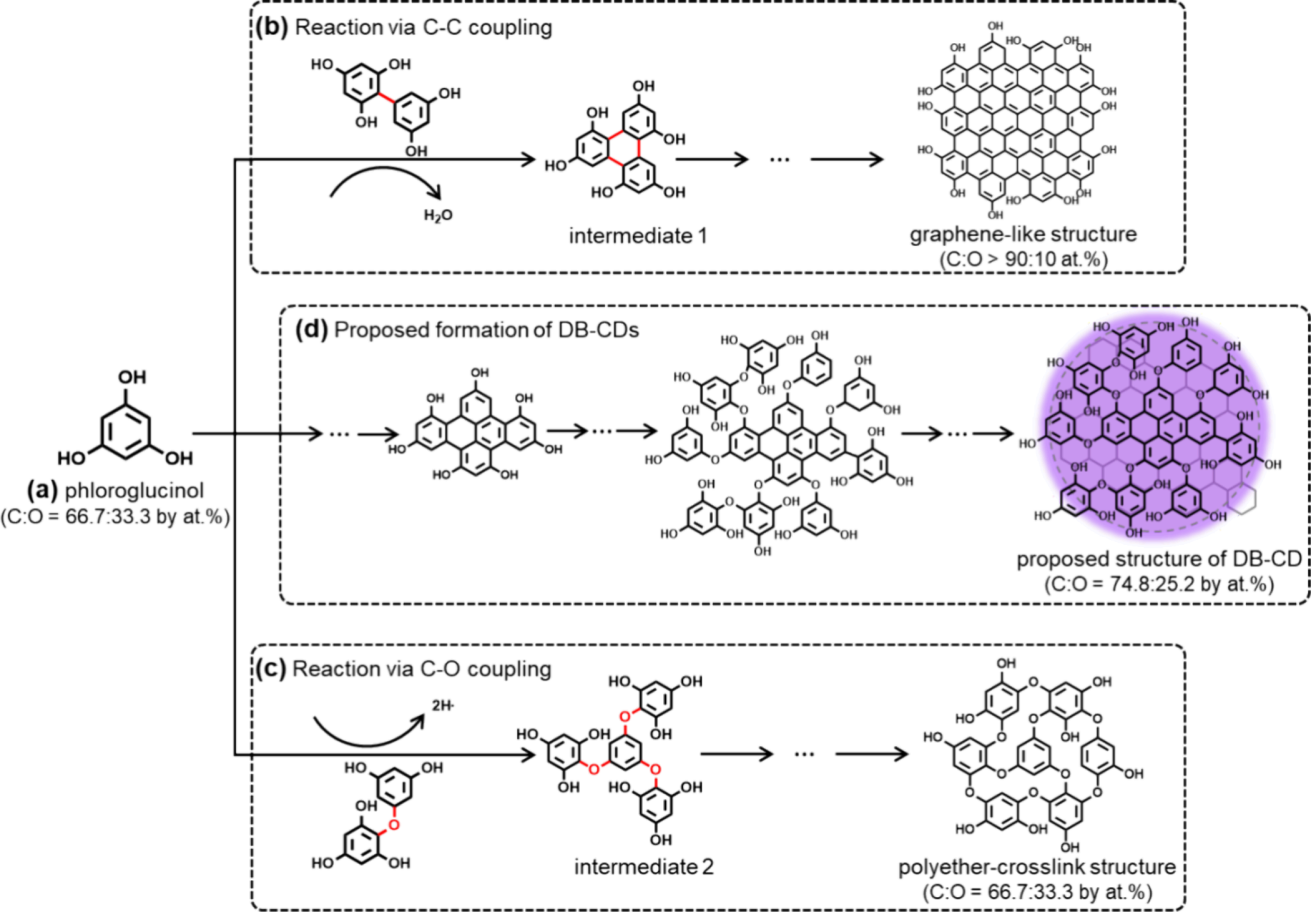
Possible chemical conversion reactions and the structure of intermediates and the final **DB-CD** product. (a) Chemical structure of the phloroglucinol precursor. (b) The C–C coupling of phloroglucinol into intermediate 1 by a trimolecular dehydration reaction and the further propagation into a graphene-like structure. (c) The C–O coupling of phloroglucinol into intermediate 2 by a trimolecular dehydrogenation reaction, and the further propagation into a polyether-cross-link structure. (d) The proposed combination of C–C and C–O coupling reactions for the formation of the **DB-CD**s, which comprise a combination of graphene segments and a polyether-cross-link network. The carbon:oxygen (C:O) atomic ratio for the different chemical structures is presented as the bottom inset.

We now turn our attention to the identification of the chemical reaction(s) that have been in effect during the conversion of the phloroglucinol precursor (chemical structure in Figure 4(a)) and the chemical structure of the final **DB-CD** product. It has been reported that the chemical conversion of phloroglucinol can follow the trimolecular dehydration pathway displayed in Figure 4(b), where the formation of three identical C–C covalent bonds between the benzene rings of three phloroglucinol molecules results in intermediate 1.^17,38,39^ The continuation of this reaction will produce a larger graphene structure, which is end-capped with phenolic-hydroxyl groups, as shown in the right part of Figure 4(b). We also call attention to that the graphene-like structure derived from such trimolecular dehydration of phloroglucinol tends to have armchair edges instead of zigzag edges.

However, analysis of the high-resolution XPS data demonstrated that the **DB-CD**s comprise a significant C–O–C component, which is lacking from the phenolic-hydroxyl-capped graphene structure depicted in the right part of Figure 4(b). Moreover, if we assume that the **DB-CD**s only comprise this phenolic-hydroxyl-capped graphene structure and that their diameter is 3.2 nm (i.e., the size of the ordered graphene structure detected by TEM in Figure 2(a)), then the carbon:oxygen (C:O) atomic ratio will be 92.3:7.7 (see Figure S4 for derivation). This is much higher than the measured C:O atomic ratio for the **DB-CD**s of 75.2:24.8, as derived from the XPS survey measurement in Figure 3(a). Thus, it is clear that the **DB-CD**s must comprise an additional carbon–oxygen chemical structure, which includes a significant C–O–C component and is relatively richer in oxygen than the graphene-like structure in Figure 4(b).

Figure 4(c) presents a second viable chemical conversion reaction of phloroglucinol, where three identical C–O–C bonds are formed between the benzene rings of three neighboring phloroglucinol molecules by a trimolecular dehydrogenation reaction for the synthesis of intermediate 2. The continuation of this reaction can result in the formation of the larger “polyether-crosslink” structure depicted in the right-hand part of Figure 4(c). It is notable that this structure does contain the “missing” C–O–C bonds but also that its C:O atomic ratio of 66.7:33.3 is lower than that measured for the **DB-CD**s.

We thus propose that the synthesis of **DB-CD**s takes place through a combination of these two chemical reactions, as schematically outlined in Figure 4(d). This implies that the **DB-CD**s comprise a combination of the distinct graphene segments and the polyether-cross-link network. It is possible to estimate their relative concentrations with the aid of the eq 5 for the carbon atomic concentration of the **DB-CD**s:^31^
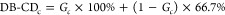
5where *G*_C_ is the relative concentration of the graphene segment, 100% is the carbon atomic concentration in an edge-group-free graphene segment, (1 *– G*_C_) is the relative concentration of the polyether-cross-link network, and 66.7% is the carbon atomic concentration in the polyether-cross-link structure. By remembering that DB-CD_C_ is equal to 74.8% (as determined by XPS), we find that the **DB-CD**s comprise 24% graphene segments and 76% polyether-like networks. Finally, the existence of hydrophilic phenolic-hydroxyl end groups on the **DB-CD**s, as depicted in Figure 4(d), is manifested in the measured high solubility of the **DB-CD**s in polar solvents, such as ethanol (8.0 g L^–1^), and the poor solubility in nonpolar solvents, such as toluene (<0.5 g L^–1^). Structural fractions that may correspond to the two proposed reaction pathways have been identified in the mass spectra (Figure S6).^40,41^

This structural assignment of **DB-CD**s is further confirmed by vibrational spectroscopy. Figure S7(a) presents the DRIFTS of **DB-CD**s dispersed in a KBr powder, with key vibrational modes marked as indicated. The broad band spanning from 3600 to 3100 cm^–1^ corresponds to O–H stretching vibrations, and the relatively sharp band cantered at 1385 cm^–1^ is attributed to C–OH symmetric stretching vibrations, both of which are assigned to the phenolic hydroxyl end groups of the **DB-CDs** (see Figure 4d).^42,43^ The highest peak at 1615 cm^–1^ is a C=C symmetric vibration within an aromatic structure, such as the graphene segments, while the strong ν(C–O–C) band at 1155 cm^–1^ can be assigned to the ether linkages in the polyether-cross-link structure.^44^Figure S7(b) shows the Raman spectrum of a ∼100 μm-thick film of **DB-CD**s on a silicon wafer. The intermediate peak at 1600 cm^–1^ is close to the characteristic G band of graphene at 1585 cm^–1^,^45^ which is in agreement with the existence of graphene segments in the **DB-CD**s.

Figure S8 describes the NMR spectra of **DB-CD**s. In the ^1^H NMR spectra, the signals in 7.5–8.0 ppm originate from aromatic protons (aromatic-H), while these in ∼6.0–7.0 ppm correspond to active hydrogen signals from hydroxy groups (−O-H).^38^ In the ^13^C NMR spectra, the signals resonating in ∼105–135 ppm are ascribed to the sp^2^ conjugated carbon atoms (aromatic-C). Additionally, the Ph-O types are detected in the range of ∼140–165 ppm, in which the signals at upfield (δ = ∼140–155 ppm) can be attributed to the carbon atoms in Ph-O-Ph structures, whereas signals at downfield (δ = ∼155–165 ppm) correspond to the carbon atoms in Ph–OH structures.^46^ The NMR data align well with the findings from the XPS and DRIFTS results, thereby further providing further support for our proposed **DB-CD** structure illustrated in Figure 4.

An important question relates to the identification of a distinct fluorophore within the **DB-CD** structure. We first performed a thorough literature study, which suggests that the polyether-cross-link structure can be excluded since such structures are reported to be optically silent in the visible range.^47−49^ In contrast, Figure S9 presents the published PL spectra of five different graphene segments,^17,50−52^ which feature PL emission in the visible wavelength range. We note with particular interest that both the C_36_H_18_ segment (Figure S9b) and the C_24_H_14_ segment (Figure S9d) feature PL peaks that are positioned in close proximity of the PL peak of the **DB-CD**s, and we thus find it plausible to tentatively assign the deep-blue emission from the **DB-CD**s to distinct graphene segments that are similar in structure to C_36_H_18_ and C_24_H_14_ and are well-dispersed into the polyether cross-link network. We have additionally identified structural fractions from the mass spectra (Figure S6) that presumably exhibit structure analogous to the C_24_H_14_ segment.

### 3.3 Application of **DB-CD**s in Random Lasing

We finally turn to an investigation of the application potential of the **DB-CD**s and begin by studying their stability under various forms of stress. Figure S10 presents the changes of the PL spectrum and the PLQY of a dry **DB-CD** powder following stress under (i) ambient air at room temperature and in darkness for 6 months, (ii) ambient air at a high temperature of 120 °C and in darkness for 24 h, and (iii) ambient air at room temperature and UV irradiation (λ_peak_ = 365 nm, power intensity = 230 W m^–2^) for 24 h. The stability test has also been implemented with DB-CDs solution, as summarized in Table S1. We find that the PL spectrum in the solid state and in solution is essentially invariant to all stress protocols and that the PLQY only dropped marginally from 51% to 46% during the most severe UV stress test. This in turn implies that the **DB-CD**s could be functional for the active material in UV-excited applications, such as the gain medium in UV-pumped random lasers.

**Figure 5:**
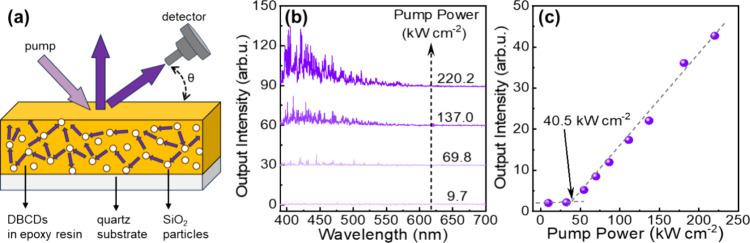
(a) Schematic illustration of the optically pumped random-laser device with emission detection at a viewing angle θ. The **DB-CD**s are the gain medium, SiO_2_ particles function as a light scattering medium, the epoxy resin is the matrix, and a quartz plate is the substrate. (b) The emission spectrum (detected at θ = 50°) of the random laser as a function of the optical pump power density (wavelength = 375 nm), as detailed above each trace. (c) The measured output intensity of the random laser as a function of the optical pump power density, with the purple circles representing measured data and the dashed purple lines two linear fits in the subthreshold region and the lasing region. The arrow indicates the established threshold optical pump power density for random lasing.

Figure 5(a) is a schematic illustration of the investigated **DB-CD** device. The **DB-CD**s are the prospective laser gain medium, the SiO_2_ particles are the scattering medium, and both these components are dispersed in an epoxy resin. This blend is spin-coated onto a quartz substrate with the dry film featuring a thickness of 700 μm. The optical pump source is a 375 nm laser, delivering pulses with a width of 6 ns. The output emission spectrum and intensity from the device are measured as a function of the viewing angle (θ).

Figure 5(b) presents the emission spectrum of the device at θ = 50° for four different values of the optical pump power density, as detailed above each emission trace while Figure 5(c) displays the integrated output intensity as a function of the optical pump power. The corresponding data for viewing angles of θ = 20° and 90° are shown in Figure S11. At low pump power, the device delivers spontaneous emission; but when the optical pump power density is increased above a threshold, being 40.5 kW cm^–2^ at θ = 50°, 61.1 kW cm^–2^ at θ = 20°, and 101.2 kW cm^–2^ at θ = 90°, the device instead emits stimulated emission, as evidenced by the emergence of sharp emission peaks and a steeper slope of the output–power vs input–power graph. This threshold is comparable with that of previously reported CDs-based lasers (see Table S2).^53−56^ The combined observations of a multitude of sharp emission peaks (see Figure 5b) and the emergence of lasing at several different viewing angles reveal that the presented device structure functions as a random laser, and – most importantly – that the **DB-CD**s can serve as the gain medium in a laser device.

## 4 Conclusions

We report on the solvothermal synthesis of **DB-CD**s using bioderivable phloroglucinol as the sole precursor and ethanol as the synthesis solvent. The synthesized **DB-CD**s exhibit notably deep-blue and narrowband emission, with a peak wavelength of 403 nm, high PLQY of 61%, and fwhm of 35 nm, and in ethanol solution. A systematic optical and structural characterization reveals that the **DB-CD**s feature a planar structure, consisting of distinct graphene segments dispersed in a polyether-cross-link network, and that it is the graphene segments that function as the fluorophores. The practical applicability of these bioderivable **DB-CD**s is demonstrated by their utilization as the gain medium in a random laser device, which exhibits a threshold optical pump power density of 40.5 kW cm^–2^. Additionally, it is worth mentioning that CDs synthesized from phloroglucinol with various PL properties have been successfully applied as active emitters and phosphors for light-emitting devices in numerous reports.^17,24^ This set of application merits represents a sustainable pathway toward the development of narrowband and high-efficiency carbon-based nanomaterials, with significant potential for advanced and sustainable optoelectronic applications.

## Supporting Information Available

CIE color coordinate, PLQY measurement of the **DB-CD**-in-ethanol solution; PL spectra of a **DB-CD** solution as a function of the solvent and of a **DB-CD** in ethanol as a function of the DB-CD concentration (or temperature); the formation of a graphene-like structure with a diameter of ∼3.2 nm; electrospray ionization mass spectra, DRIFTS, Raman, and NMR spectra of **DB-CD**s; PL spectra of graphene segments; PL stability of **DB-CD**s in solid state and solution state; emission spectrum of the **DB-CD** random laser as a function of the optical pump power at two different viewing angles (θ = 20° and θ = 90°), survey of CD-based lasing (PDF)

## References

[ref1] Han P.; Xia E.; Qin A.; Tang B. Z. Adjustable and smart AIEgens for nondoped blue and deep blue organic light-emitting diodes. Coord. Chem. Rev. 2022, 473, 214843 10.1016/j.ccr.2022.214843.

[ref2] Yang X.; Xu X.; Zhou G. Recent advances of the emitters for high performance deep-blue organic light-emitting diodes. J. Mater. Chem. C 2015, 3, 913–944. 10.1039/C4TC02474E.

[ref3] Zhang W.; Li B.; Chang C.; Chen F.; Zhang Q.; Lin Q.; Wang L.; Yan J.; Wang F.; Chong Y.; Du Z.; Fan F.; Shen H. Stable and efficient pure blue quantum-dot LEDs enabled by inserting an anti-oxidation layer. Nat. Commun. 2024, 15, 783 10.1038/s41467-024-44894-z. 38278797 PMC10817946

[ref4] Chen X.; Lin X.; Zhou L.; Sun X.; Li R.; Chen M.; Yang Y.; Hou W.; Wu L.; Cao W.; Zhang X.; Yan X.; Chen S. Blue light-emitting diodes based on colloidal quantum dots with reduced surface-bulk coupling. Nat. Commun. 2023, 14, 284 10.1038/s41467-023-35954-x. 36650161 PMC9845229

[ref5] Jang K. Y.; Hwang S. Y.; Woo S.-J.; Yoon E.; Park C.-Y.; Kim S. Y.; Kim D.-H.; Kim H.; Park J.; Sargent E. H.; Lee T.-W. Efficient deep-blue light-emitting diodes through decoupling of colloidal perovskite quantum dots. Adv. Mater. 2024, 36, 2404856 10.1002/adma.202404856. 39109569

[ref6] Shen W.; Qiu Y.; Jiang J.; Chen Z.; He Y.; Cui H.; Liu L.; Cheng G.; Aleshin A. N.; Chen S. Stable deep-blue FAPbBr_3_ quantum dots facilitated by amorphous metal halide matrices. Chem. Commun. 2023, 59, 11137–11140. 10.1039/D3CC03415A. 37650131

[ref7] Saha A.; Yadav R.; Aldakov D.; Reiss P. Gallium sulfide quantum dots with zinc sulfide and alumina shells showing efficient deep blue emission. Angew. Chem., Int. Ed. 2023, 62, e202311317 10.1002/anie.202311317. 37735098

[ref8] Guo Q.; Wang L.; Yang L.; Duan J.; Du H.; Ji G.; Liu N.; Zhao X.; Chen C.; Xu L.; Gao L.; Luo J.; Tang J. Spectra stable deep-blue light-emitting diodes based on cryolite-like cerium(III) halides with nanosecond d-f emission. Sci. Adv. 2022, 8, eabq2148 10.1126/sciadv.abq2148. 36525491 PMC9757739

[ref9] Feng Z.; Cheng Z.; Su Z.; Wan L.; Ge S.; Liu H.; Ma X.; Liu F.; Lu P. Realizing efficient deep blue light-emitting diodes and single component white light-emitting diodes with low efficiency roll-offs from anthracene-based organic emitters. Adv. Opt. Mater. 2024, 12, 2302839 10.1002/adom.202302839.

[ref10] Xu Y.; Liang X.; Liang Y.; Guo X.; Hanif M.; Zhou J.; Zhou X.; Wang C.; Yao J.; Zhao R.; Hu D.; Qiao X.; Ma D.; Ma Y. Efficient deep-blue fluorescent OLEDs with a high exciton utilization efficiency from a fully twisted phenanthroimidazole–anthracene emitter. ACS Appl. Mater. Interfaces 2019, 11, 31139–31146. 10.1021/acsami.9b10823. 31368304

[ref11] Wang S.; Sun L.; Zheng Y.; Zhang Y.; Yu N.; Yang J.; Li M.; Chen W.; He L.; Liu B.; Ni M.; Liu H.; Xu M.; Bai L.; Lin J.; Huang W. Large-area blade-coated deep-blue polymer light-emitting diodes with a narrowband and uniform emission. Adv. Sci. 2023, 10, 2205411 10.1002/advs.202205411. PMC9951302 36574468

[ref12] Liu B.; He L.; Li M.; Yu N.; Chen W.; Wang S.; Sun L.; Ni M.; Bai L.; Pan W.; Sun P.; Lin J.; Huang W. Improving the intrinsic stretchability of fully conjugated polymer for deep-blue polymer light-emitting diodes with a narrow band emission: benefits of self-toughness effect. J. Phys. Chem. Lett. 2022, 13, 7286–7295. 10.1021/acs.jpclett.2c02071. 35916779

[ref13] Ren J.; Opoku H.; Tang S.; Edman L.; Wang J. Carbon dots: A review with focus on sustainability. Adv. Sci. 2024, 11, 2405472 10.1002/advs.202405472. PMC11425242 39023174

[ref14] Ren J.; Stagi L.; Innocenzi P. Fluorescent carbon dots in solid-state: From nanostructures to functional devices. Prog. Solid State Chem. 2021, 62, 100295 10.1016/j.progsolidstchem.2020.100295.

[ref15] Liu J.; Geng Y.; Li D.; Yao H.; Huo Z.; Li Y.; Zhang K.; Zhu S.; Wei H.; Xu W.; Jiang J.; Yang B. Deep red emissive carbonized polymer dots with unprecedented narrow full width at half maximum. Adv. Mater. 2020, 32, 1906641 10.1002/adma.201906641. 32191372

[ref16] Tang S.; Liu Y.; Opoku H.; Gregorsson M.; Zhang P.; Auroux E.; Dang D.; Mudring A.-V.; Wagberg T.; Edman L.; Wang J. Fluorescent carbon dots from birch leaves for sustainable electroluminescent devices. Green Chem. 2023, 25, 9884–9895. 10.1039/D3GC03827K.

[ref17] Yuan F.; Yuan T.; Sui L.; Wang Z.; Xi Z.; Li Y.; Li X.; Fan L.; Tan Z.; Chen A.; Jin M.; Yang S. Engineering triangular carbon quantum dots with unprecedented narrow bandwidth emission for multicolored LEDs. Nat. Commun. 2018, 9, 2249 10.1038/s41467-018-04635-5. 29884873 PMC5993800

[ref18] Kalytchuk S.; Zdražil L.; Scheibe M.; Zbořil R. Purple-emissive carbon dots enhance sensitivity of Si photodetectors to ultraviolet range. Nanoscale 2020, 12, 8379–8384. 10.1039/D0NR00505C. 32239023

[ref19] Yuan F.; Wang Y.-K.; Sharma G.; Dong Y.; Zheng X.; Li P.; Johnston A.; Bappi G.; Fan J. Z.; Kung H.; Chen B.; Saidaminov M. I.; Singh K.; Voznyy O.; Bakr O. M.; Lu Z.-H.; Sargent E. H. Bright high-colour-purity deep-blue carbon dot light-emitting diodes via efficient edge amination. Nat. Photonics 2020, 14, 171–176. 10.1038/s41566-019-0557-5.

[ref20] Wang X.; Zhang X.; Gu X.; Nie H.; Zhu M.; Wang B.; Gao J.; Tao Y.; Zhu Y.; Huang H.; Xu C.; Shao M.; Liu Y.; Liao L.; Kang Z. A bright and stable violet carbon dot light-emitting diode. Adv. Opt. Mater. 2020, 8, 2000239 10.1002/adom.202000239.

[ref21] Huang P.; Li M.-Z.; Wen C.-F.; Zhou H.-Y.; Jian J.-X.; Tong Q.-X. Nitrogen-doped carbon dots for efficient deep-blue light-emitting diodes with CIE closely approaching the HDTV standard color Rec.BT.709. Chem. Commun. 2023, 59, 8933–8936. 10.1039/D3CC02105J. 37401807

[ref22] Wang B.; Wang H.; Zhang B.; Hu Y.; Lu S. Hot exciton carbon dots-based deep blue electroluminescent light-emitting diodes exceeding 7% external quantum efficiency. Adv. Funct. Mater. 2024, 34, 2404437 10.1002/adfm.202404437.

[ref23] Zhao B.; Ma H.; Zheng M.; Xu K.; Zou C.; Qu S.; Tan Z. a. Narrow-bandwidth emissive carbon dots: A rising star in the fluorescent material family. Carbon Energy 2022, 4, 88–114. 10.1002/cey2.175.

[ref24] Liu Y.; Tang S.; Wu X.; Boulanger N.; Gracia-Espino E.; Wågberg T.; Edman L.; Wang J. Carbon nanodots: A metal-free, easy-to-synthesize, and benign emitter for light-emitting electrochemical cells. Nano Res. 2022, 15, 5610–5618. 10.1007/s12274-022-4126-8.

[ref25] Xie Y.; Peng B.; Bravić I.; Yu Y.; Dong Y.; Liang R.; Ou Q.; Monserrat B.; Zhang S. Highly efficient blue-emitting CsPbBr_3_ perovskite nanocrystals through neodymium doping. Adv. Sci. 2020, 7, 2001698 10.1002/advs.202001698. PMC7578857 33101870

[ref26] Kim H. J.; Kang H.; Jeong J.-E.; Park S. H.; Koh C. W.; Kim C. W.; Woo H. Y.; Cho M. J.; Park S.; Choi D. H. Ultra-deep-blue aggregation-induced delayed fluorescence emitters: Achieving nearly 16% EQE in solution-processed nondoped and doped oleds with CIE_y_ < 0.1. Adv. Funct. Mater. 2021, 31, 2102588 10.1002/adfm.202102588.

[ref27] Ha J. M.; Hur S. H.; Pathak A.; Jeong J.-E.; Woo H. Y. Recent advances in organic luminescent materials with narrowband emission. NPG Asia Mater. 2021, 13, 53 10.1038/s41427-021-00318-8.

[ref28] Wang Z.; Dong X.; Zhou S.; Xie Z.; Zalevsky Z. Ultra-narrow-bandwidth graphene quantum dots for superresolved spectral and spatial sensing. NPG Asia Mater. 2021, 13, 5 10.1038/s41427-020-00269-6.

[ref29] Jang M.-H.; Song S. H.; Ha H. D.; Seo T. S.; Jeon S.; Cho Y.-H. Origin of extraordinary luminescence shift in graphene quantum dots with varying excitation energy: An experimental evidence of localized sp^2^ carbon subdomain. Carbon 2017, 118, 524–530. 10.1016/j.carbon.2017.03.060.

[ref30] Yoon H.; Chang Y. H.; Song S. H.; Lee E.-S.; Jin S. H.; Park C.; Lee J.; Kim B. H.; Kang H. J.; Kim Y.-H.; Jeon S. Intrinsic photoluminescence emission from subdomained graphene quantum dots. Adv. Mater. 2016, 28, 5255–5261. 10.1002/adma.201600616. 27153519

[ref31] Ren J.; Ye K.; Opoku H.; Li Z.; Edman L.; Wang J. Controlling the emission colour and chemical structure of carbon dots by catalysis-tuned conversion of ortho-aminophenol. Carbon 2025, 231, 119706 10.1016/j.carbon.2024.119706.

[ref32] Empirical Parameters of Solvent Polarity. In Solvents and Solvent Effects in Organic Chemistry, 4th ed.; Reichardt C.; Welton T., Eds. John Wiley & Sons: 2010; pp 425–508.

[ref33] Wang L.; Wang Y.; Xu T.; Liao H.; Yao C.; Liu Y.; Li Z.; Chen Z.; Pan D.; Sun L.; Wu M. Gram-scale synthesis of single-crystalline graphene quantum dots with superior optical properties. Nat. Commun. 2014, 5, 5357 10.1038/ncomms6357. 25348348

[ref34] Park J.-S.; Lee M.-H.; Jeon I.-Y.; Park H.-S.; Baek J.-B.; Song H.-K. Edge-exfoliated graphites for facile kinetics of delithiation. ACS Nano 2012, 6, 10770–10775. 10.1021/nn3050227. 23189955

[ref35] Li P.; Xue S.; Sun L.; Zong X.; An L.; Qu D.; Wang X.; Sun Z. Formation and fluorescent mechanism of red emissive carbon dots from o-phenylenediamine and catechol system. Light: Sci. Appl. 2022, 11, 298 10.1038/s41377-022-00984-5. 36229434 PMC9561683

[ref36] Kovtun A.; Jones D.; Dell’Elce S.; Treossi E.; Liscio A.; Palermo V. Accurate chemical analysis of oxygenated graphene-based materials using X-ray photoelectron spectroscopy. Carbon 2019, 143, 268–275. 10.1016/j.carbon.2018.11.012.

[ref37] Major G. H.; Fairley N.; Sherwood P. M. A.; Linford M. R.; Terry J.; Fernandez V.; Artyushkova K. Practical guide for curve fitting in X-ray photoelectron spectroscopy. J. Vac. Sci. Technol., A 2020, 38, 061203 10.1116/6.0000377.

[ref38] Dutta S. D.; Hexiu J.; Kim J.; Sarkar S.; Mondal J.; An J. M.; Lee Y.-k.; Moniruzzaman M.; Lim K.-T. Two-photon excitable membrane targeting polyphenolic carbon dots for long-term imaging and pH-responsive chemotherapeutic drug delivery for synergistic tumor therapy. Biomater. Sci. 2022, 10, 1680–1696. 10.1039/D1BM01832A. 35147614

[ref39] Yuan F.; Xi Z.; Shi X.; Li Y.; Li X.; Wang Z.; Fan L.; Yang S. Ultrastable and low-threshold random lasing from narrow-bandwidth-emission triangular carbon quantum dots. Adv. Opt. Mater. 2019, 7, 1801202 10.1002/adom.201801202.

[ref40] Ahmad M. A.; Sumarsih S.; Chang J.-y.; Fahmi M. Z. Mass spectrometry-based analyses of carbon nanodots: Structural elucidation. ACS Omega 2024, 9, 20720–20727. 10.1021/acsomega.4c01674. 38764670 PMC11097173

[ref41] Hu Q.; Meng X.; Chan W. An investigation on the chemical structure of nitrogen and sulfur codoped carbon nanoparticles by ultra-performance liquid chromatography-tandem mass spectrometry. Anal. Bioanal. Chem. 2016, 408, 5347–5357. 10.1007/s00216-016-9631-8. 27225175

[ref42] Zhang C.; Dabbs D. M.; Liu L.-M.; Aksay I. A.; Car R.; Selloni A. Combined effects of functional groups, lattice defects, and edges in the infrared spectra of graphene oxide. J. Phys. Chem. C 2015, 119, 18167–18176. 10.1021/acs.jpcc.5b02727.

[ref43] Ren J.; Qu D.; Liu J.; Wei B.; Innocenzi P. Observing carbon dots in putative boron nitride quantum dots. Nano Lett. 2024, 24, 15444–15449. 10.1021/acs.nanolett.4c05001. 39564982

[ref44] Coates J. Interpretation of infrared spectra, a practical approach. In Encyclopedia of Analytical Chemistry, Meyers R. A., Ed. John Wiley & Sons Ltd.: 2000; pp 10881–10882.

[ref45] Fois L.; Stagi L.; Carboni D.; Alboushi M.; Khaleel A.; Anedda R.; Innocenzi P. The formation of carbon dots from D-glucose studied by infrared spectroscopy. Chem. - Eur. J. 2024, 30, e202400158 10.1002/chem.202400158. 38619533

[ref46] Gohda S.; Saito M.; Yamada Y.; Kanazawa S.; Ono H.; Sato S. Carbonization of phloroglucinol promoted by heteropoly acids. J. Mater. Sci. 2021, 56, 2944–2960. 10.1007/s10853-020-05393-w.

[ref47] Koike R.; Katayose Y.; Ohta A.; Motoyoshiya J.; Nishii Y.; Aoyama H. Poly(benzyl ether) dendrimers with strongly fluorescent distyrylbenzene cores as the fluorophores for peroxyoxalate chemiluminescence: insulating effect of dendritic structures on fluorescent sites. Tetrahcdron 2005, 61, 11020–11026. 10.1016/j.tet.2005.08.108.

[ref48] Kimata S.-I.; Jiang D.-L.; Aida T. Morphology-dependent luminescence properties of poly(benzyl ether) dendrimers. J. Polym. Sci., Part A: Polym. Chem. 2003, 41, 3524–3530. 10.1002/pola.10830.

[ref49] Ortiz A. M.; Sánchez-Méndez A.; de Jesús E.; Flores J. C.; Gómez-Sal P.; Mendicuti F. Poly(benzyl ether) dendrimers functionalized at the core with palladium bis(N-heterocyclic carbene) complexes as catalysts for the heck coupling reaction. lnorg. Chem. 2016, 55, 1304–1314. 10.1021/acs.inorgchem.5b02629. 26788881

[ref50] Rietsch P.; Soyka J.; Brülls S.; Er J.; Hoffmann K.; Beerhues J.; Sarkar B.; Resch-Genger U.; Eigler S. Fluorescence of a chiral pentaphene derivative derived from the hexabenzocoronene Motif. Chem. Commun. 2019, 55, 10515–10518. 10.1039/C9CC05451K. 31414103

[ref51] Schoental R.; Scott E. J. Y. 362. Fluorescence spectra of polycyclic aromatic hydrocarbons in solution. Journal of the Chemical Society (Resumed) 1949, 1683–1696. 10.1039/jr9490001683.

[ref52] Taniguchi M.; Lindsey J. S. Database of absorption and fluorescence spectra of > 300 common compounds for use in PhotochemCAD. Photochem. Photobiol. 2018, 94, 290–327. 10.1111/php.12860. 29166537

[ref53] Madonia A.; Minervini G.; Terracina A.; Pramanik A.; Martorana V.; Sciortino A.; Carbonaro C. M.; Olla C.; Sibillano T.; Giannini C.; Fanizza E.; Curri M. L.; Panniello A.; Messina F.; Striccoli M. Dye-derived red-emitting carbon dots for lasing and solid-state lighting. ACS Nano 2023, 17, 21274–21286. 10.1021/acsnano.3c05566. 37870465 PMC10655242

[ref54] Han Z.; Ni Y.; Ren J.; Zhang W.; Wang Y.; Xie Z.; Zhou S.; Yu S. F. Highly efficient and ultra-narrow bandwidth orange emissive carbon dots for microcavity lasers. Nanoscale 2019, 11, 11577–11583. 10.1039/C9NR03448J. 31169274

[ref55] Wang J.; Zhang S.; Li Y.; Wu C.; Zhang W.; Zhang H.; Xie Z.; Zhou S. Ultra-broadband random laser and white-light emissive carbon dots/crystal in-situ hybrids. Small 2022, 18, 2203152 10.1002/smll.202203152. 36026553

[ref56] Zhang Y.; Hu Y.; Lin J.; Fan Y.; Li Y.; Lv Y.; Liu X. Excitation wavelength independence: Toward low-threshold amplified spontaneous emission from carbon nanodots. ACS Appl. Mater. Interfaces 2016, 8, 25454–25460. 10.1021/acsami.6b08315. 27617695

