# Supplementary material for: Deep-Blue and Narrowband-Emitting Carbon Dots from a Sustainable Precursor for Random Lasing
Source: ACS Appl Nano Mater. 2025 Jan 30;8(5):2472–80. doi: 10.1021/acsanm.4c06734 (PMC11811926; doi:10.1021/acsanm.4c06734)
Supplement: Supplementary file 1 — an4c06734_si_001.pdf [file an4c06734_si_001.pdf]

Supporting Information

Deep-Blue and Narrowband Emitting Carbon Dots from a Sustainable Precursor for Random Lasing

Junkai Ren[a,c], Jiong Liu[b], Bing Wei[c], Wenfei Zhang[b,], Ludvig Edman[a,d]*, Jia Wang[a,d]**

[a] The Organic Photonics and Electronics Group, Department of Physics, Umeå University, SE-90187 Umeå, Sweden
E-mail: ludvig.edman@umu.se, jia.wang@umu.se

[b] Key Laboratory of Optoelectronic Devices and Systems of Ministry of Education and Guangdong Province, College of Physics and Optoelectronic Engineering, Shenzhen University, 518060 Shenzhen, China
E-mail: zhangwf@szu.edu.cn

[c] School of Physics, Xidian University, 710071 Xi'an, China

[d] Wallenberg Initiative Materials Science for Sustainability, Department of Physics, Umeå University, SE-90187 Umeå, Sweden

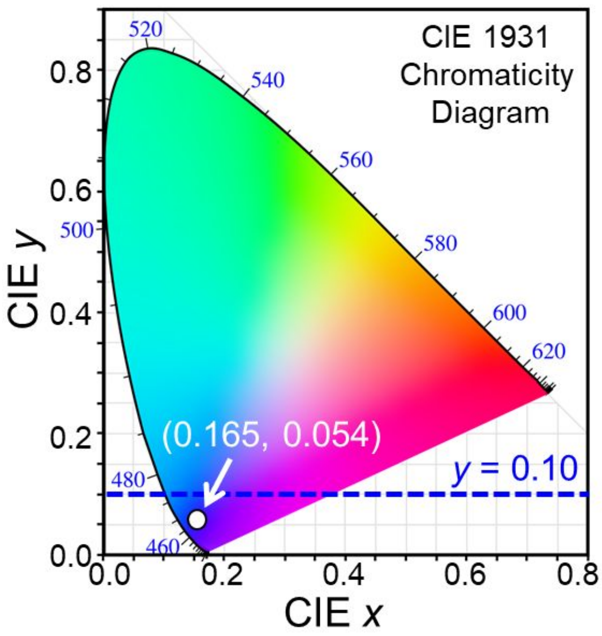

Figure S1. *The color coordinates of the PL spectrum of the* ***DB-CD****-in-ethanol solution in the CIE 1931 diagram.*

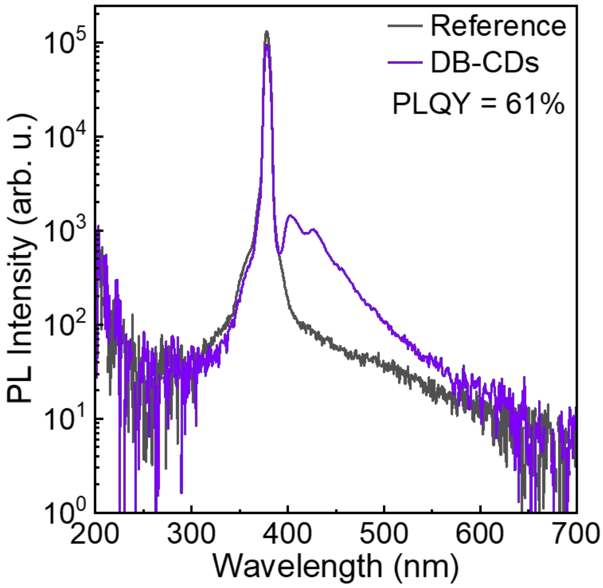

Figure S2. *The PLQY measurement of the* ***DB-CD****-in-ethanol solution by an absolute method. The excitation wavelength is 360 nm.*

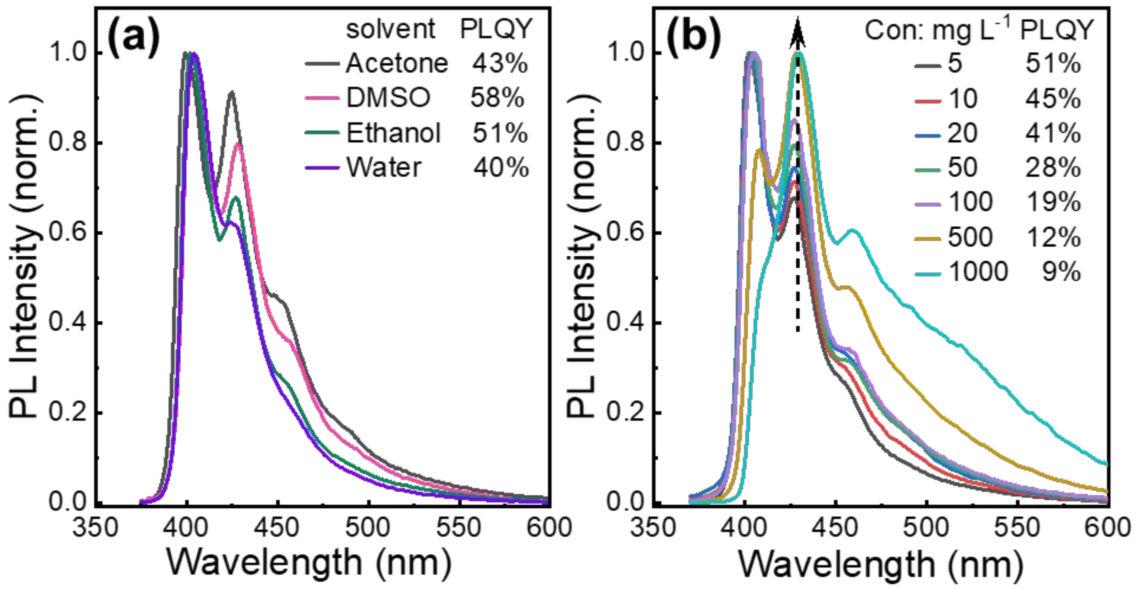

Figure S3. ***(a)*** *The normalized PL spectrum and the PLQY (inset) of a* ***DB-CD*** *solution as a function of the solvent, as specified in the inset. The solute concentration is 10 mg* L^{-1}. ***(b)*** *The normalized PL spectrum and the PLQY (inset) of* ***DB-CDs*** *in ethanol solution as a function of the* ***DB-CD*** *concentration, as specified in the inset. The arrow indicates increasing concentration. The excitation wavelength is 360 nm.*

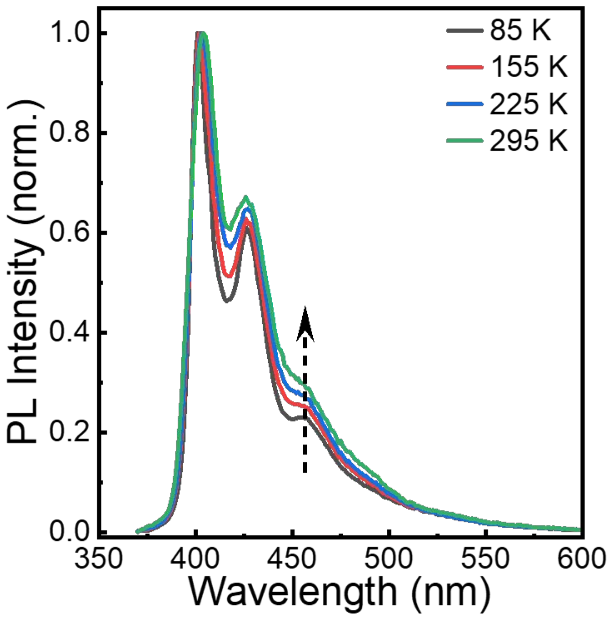

Figure S4. *The normalized temperature-dependent PL spectra of* ***DB-CDs*** *in ethanol solution. The arrow indicates increasing temperature, and the excitation wavelength is 360 nm.*

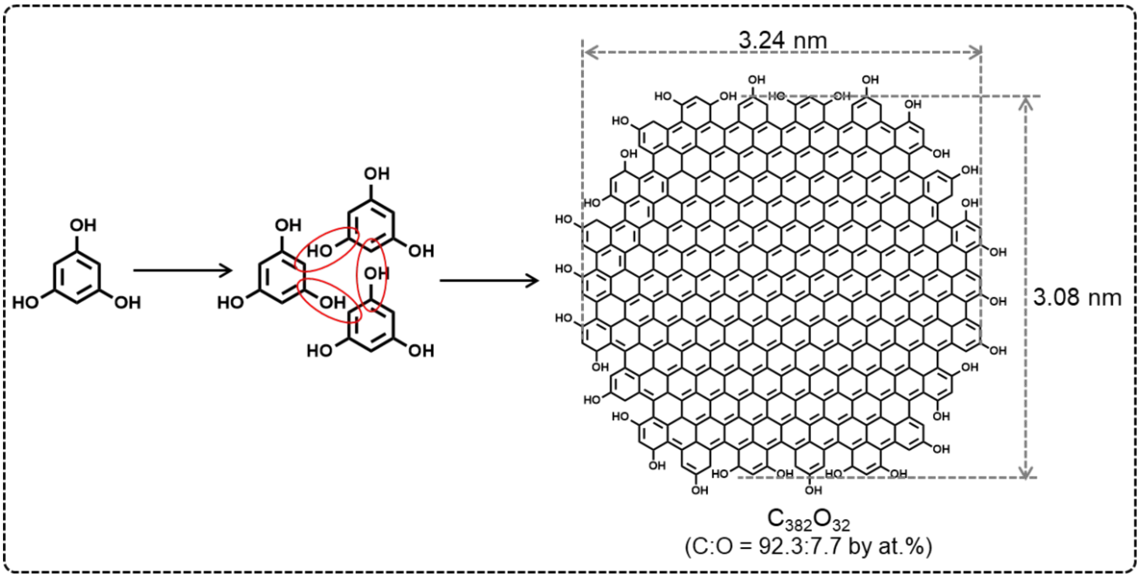

Figure S5. *The chemical conversion of the phloroglucinol precursor (left panel) by a C-C coupling tri-molecular dehydration reaction (middle panel), which ends with the formation of a graphene-like structure with a diameter of ≈ 3.2 nm (right panel).*

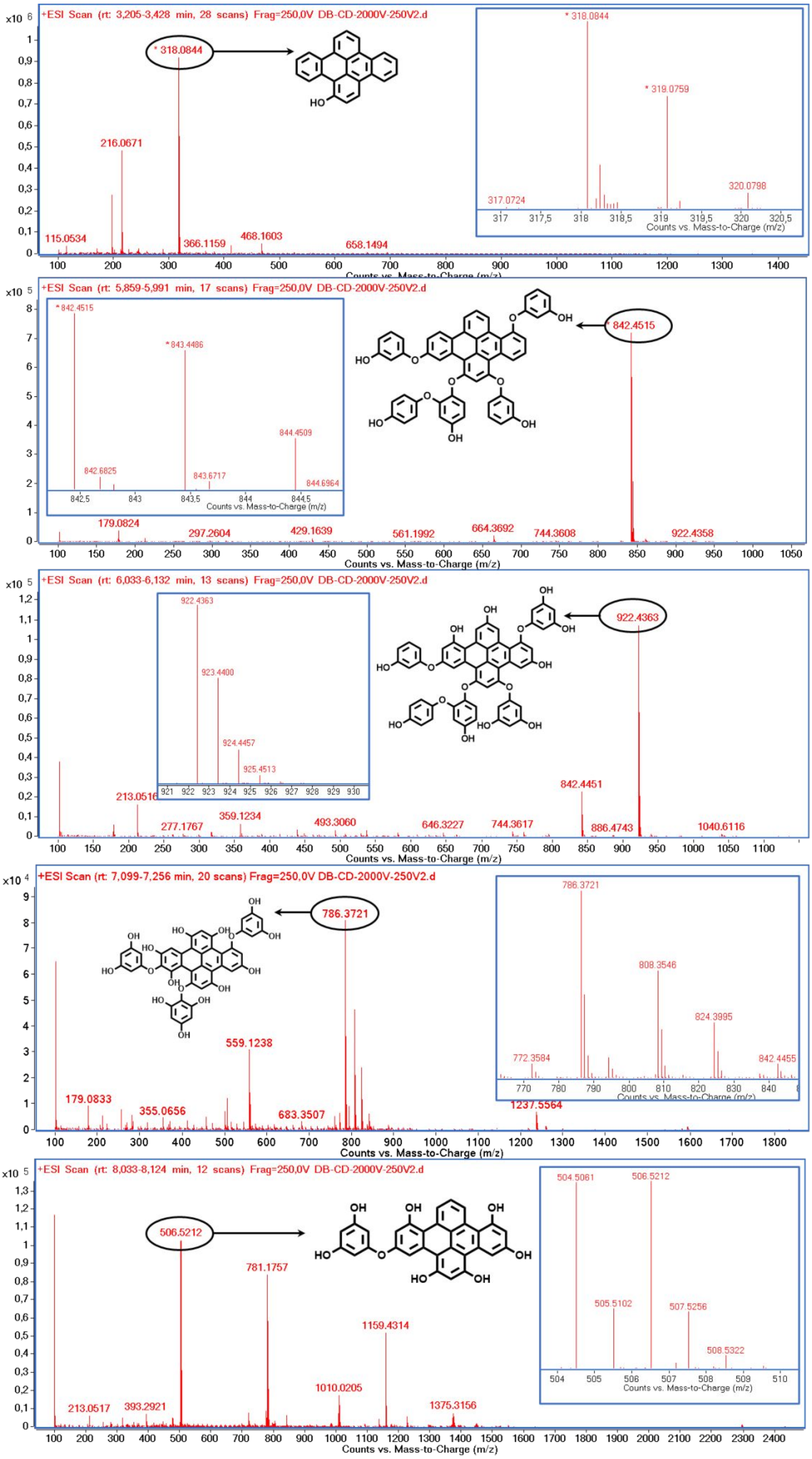

Figure S6. *Electrospray ionization mass spectra.*

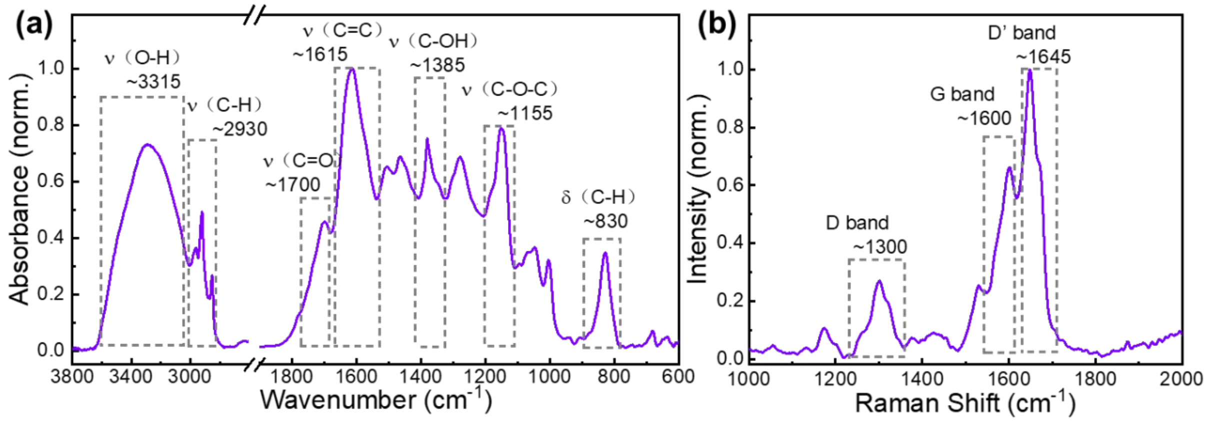

Figure S7. (a) *The normalized DRIFTS spectrum of* ***DB-CDs*** *dispersed in a KBr powder, with key vibrational modes identified by the dashed grey rectangles.* ***(b)*** *The normalized Raman spectrum of a ~100 μm thick film of* ***DB-CDs*** *on a silicon wafer, with key bands marked by dashed grey rectangles.*

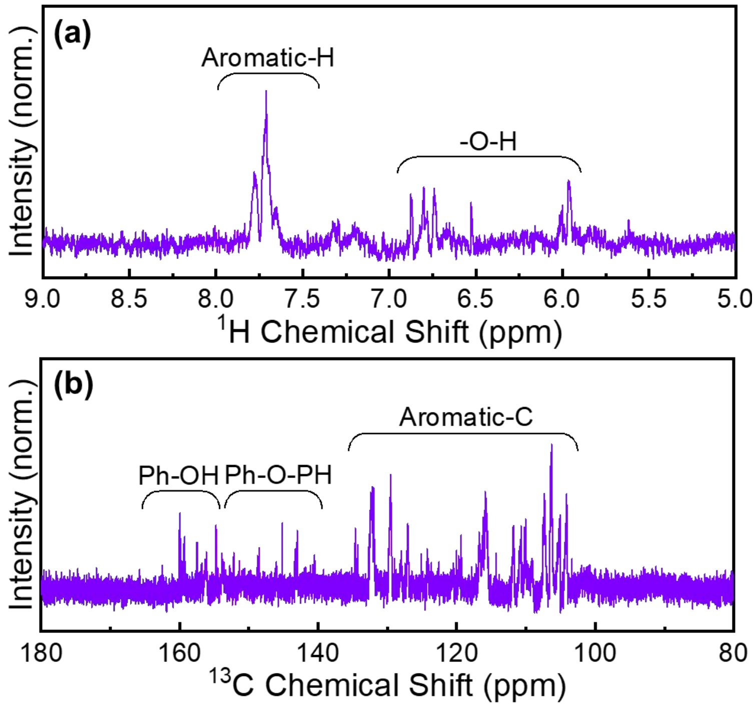

Figure S8*.* ***(a)*** *The ^{1}H NMR spectrum in DMSO-d_6 and* ***(b)*** *^{13}C NMR spectrum in methanol-d_4 of* ***DB-CDs****.*

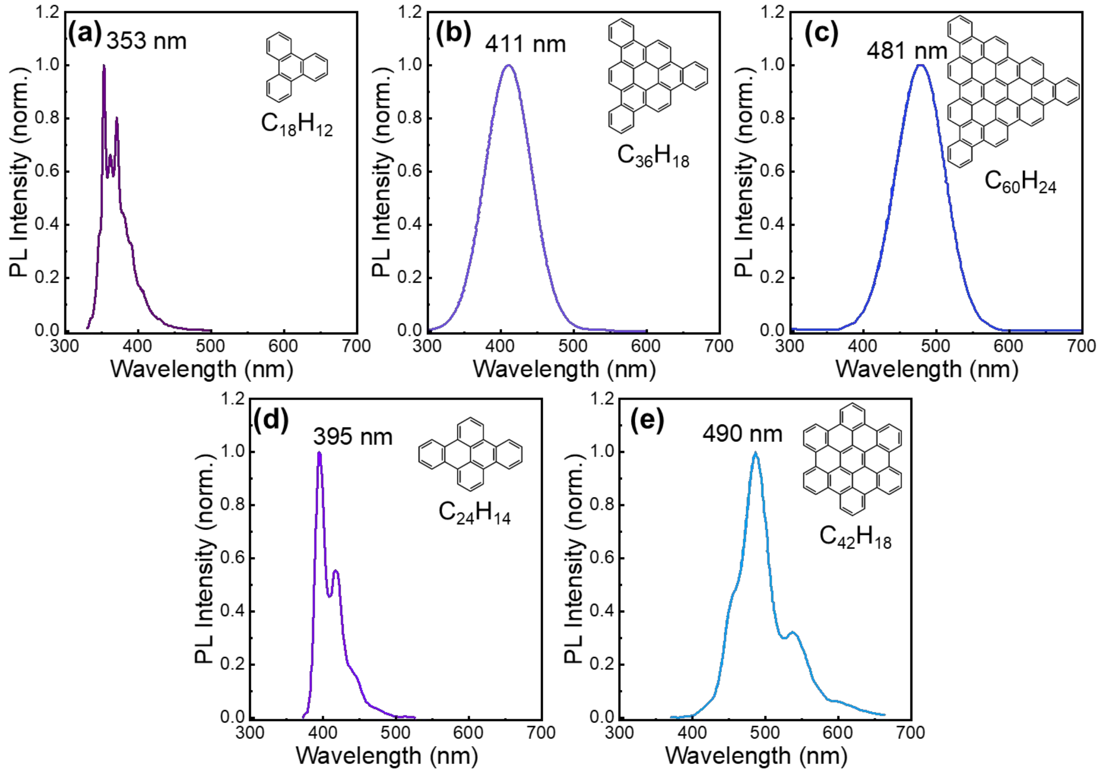

Figure S9. *Literature-derived PL spectra of five different graphene segments:* ***(a)*** *$C_{18}H_{12}$ (measured in Ref.* [1]*),* ***(b)*** *$C_{36}H_{18}$ and* ***(c)*** *$C_{60}H_{24}$, (simulated in Ref.* [2]*)* ***(d)*** *$C_{24}H_{14}$ measured in Ref.*[3]*, and* ***(e)*** *$C_{42}H_{18}$ measured in Ref.*[4]*.*

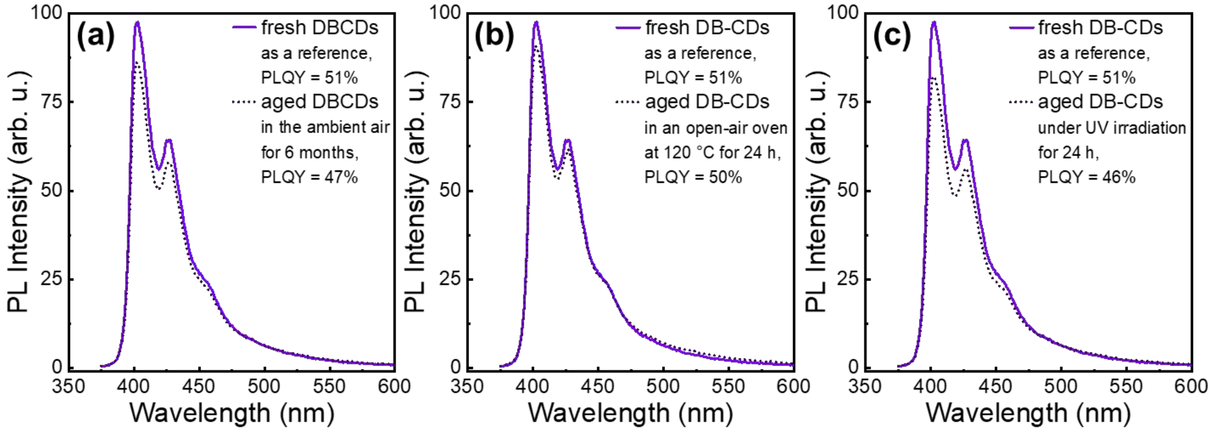

Figure S10. *The PL stability of dry* ***DB-CDs*** *under various stress conditions. The dry* ***DB-CDs*** *were subjected to storage* ***(a)*** *in darkness under ambient air for 6 months,* ***(b)*** *in darkness at 120 °C under ambient air for 24 h, or* ***(c)*** *under exposure to UV light (λ_{peak} = 365 nm, power intensity = 230 W m^{-2}) under ambient air for 24 h. After the stress treatment, the* ***DB-CDs*** *were re-dispersed in ethanol, and the PL spectrum measurement (dotted lines). The PL spectrum of a pristine* ***DB-CD*** *in ethanol solution (solid lines) were included as reference. The solute concentration was 1.0 mg L^{-1}, and the excitation wavelength was 360 nm.*

***Table S1.** The PL stability of **DB-CD**-in-ethanol solution under various stress conditions. The solute concentration was 1.0 mg L^{-1}, and the excitation wavelength was 360 nm.*

Samples	PLQY
Fresh **DB-CD**-in-ethanol solution	51%
**DB-CD**-in-ethanol solution (closed in a vial and in darkness at 120 °C for 24 h)	49%
**DB-CD**-in-ethanol solution (closed in a vial and under exposure to UV light (λ_{peak} = 365 nm, power intensity = 230 W m^{-2} for 24 h)	48%

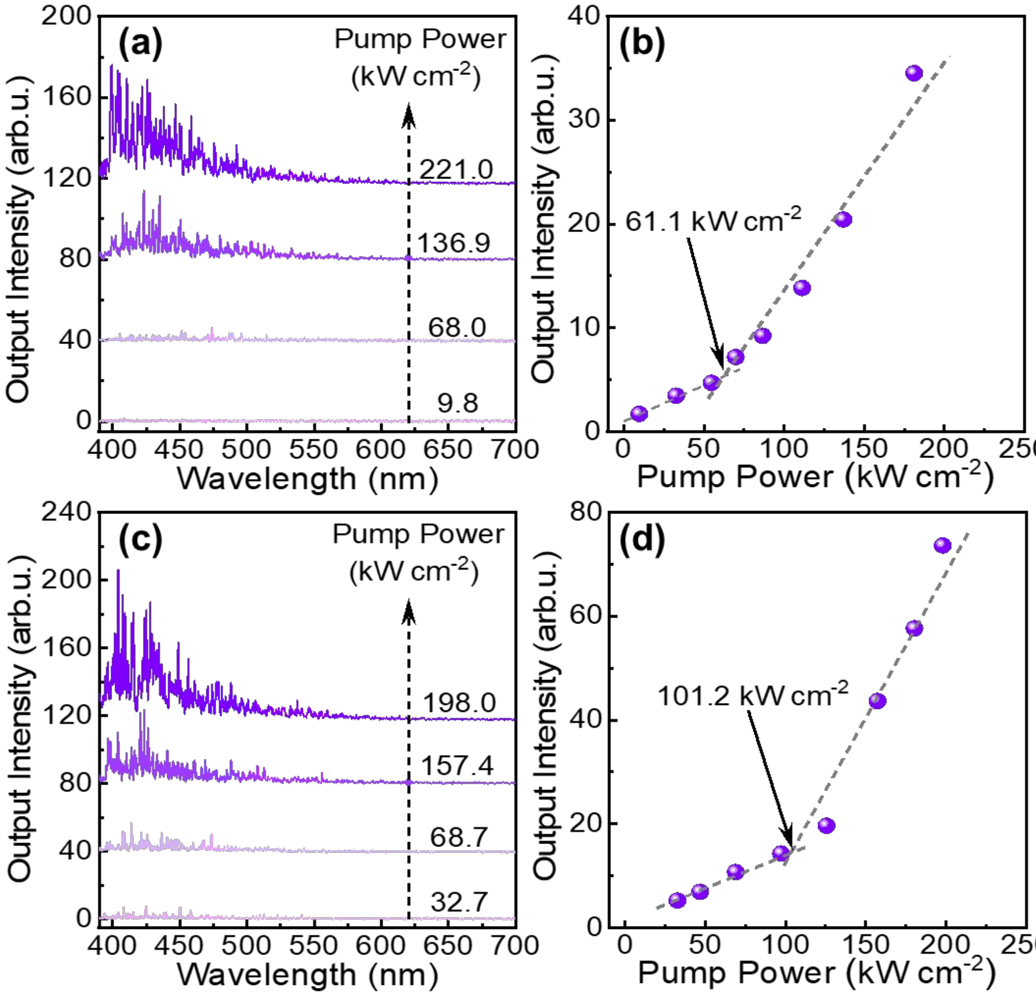

***Figure S11. (a,c)** The emission spectrum of the **DB-CD** random laser as a function of the optical pump power at two different viewing angles (**a**, θ = 20°; **c**, θ = 90°). The excitation wavelength was λ = 375 nm, the optical pump power is indicated above each trace, and the arrow indicates increasing pump power. **(b,d)** The output intensity of the **DB-CD** random laser as a function of the optical pump power at two different viewing angles (**b**, θ = 20°; **d**, θ = 90°). The purple dots are the measured data, and the grey dashed lines are the result of a linear fitting. The arrows indicate the threshold for the optical pump power density.*

Table S2. *Survey of CDs-based lasing: optical properties and lasing threshold.*

PL Peak	FWHM	PLQY	lasing threshold	Ref.
630 nm	-	7.0%	100 kW cm^{-2}	[5]
581 nm	30 nm	82%	12 kW cm^{-2}	[6]
530 nm	-	21%	60 kW cm^{-2}	[7]
458 nm	70 nm	82%	80 kW cm^{-2}	[8]
403 nm	35 nm	61%	40.5 kW cm^{-2}	This work

References

(1) Taniguchi, M.; Lindsey, J. S., Database of absorption and fluorescence spectra of >300 common compounds for use in PhotochemCAD. *Photochem. Photobiol.* **2018,** *94*, 290-327.

(2) Yuan, F.; Yuan, T.; Sui, L.; Wang, Z.; Xi, Z.; Li, Y.; Li, X.; Fan, L.; Tan, Z.; Chen, A.; Jin, M.; Yang, S., Engineering triangular carbon quantum dots with unprecedented narrow bandwidth emission for multicolored LEDs. *Nat. Commun.* **2018,** *9*, 2249.

(3) Schoental, R.; Scott, E. J. Y., 362. Fluorescence spectra of polycyclic aromatic hydrocarbons in solution. *Journal of the Chemical Society (Resumed)* **1949**, 1683-1696.

(4) Rietsch, P.; Soyka, J.; Brülls, S.; Er, J.; Hoffmann, K.; Beerhues, J.; Sarkar, B.; Resch-Genger, U.; Eigler, S., Fluorescence of a chiral pentaphene derivative derived from the hexabenzocoronene Motif. *Chem. Commun.* **2019,** *55*, 10515-10518.

(5) Madonia, A.; Minervini, G.; Terracina, A.; Pramanik, A.; Martorana, V.; Sciortino, A.; Carbonaro, C. M.; Olla, C.; Sibillano, T.; Giannini, C.; Fanizza, E.; Curri, M. L.; Panniello, A.; Messina, F.; Striccoli, M., Dye-derived red-emitting carbon dots for lasing and solid-state lighting. *ACS Nano* **2023,** *17*, 21274-21286.

(6) Han, Z.; Ni, Y.; Ren, J.; Zhang, W.; Wang, Y.; Xie, Z.; Zhou, S.; Yu, S. F., Highly efficient and ultra-narrow bandwidth orange emissive carbon dots for microcavity lasers. *Nanoscale* **2019,** *11*, 11577-11583.

(7) Wang, J.; Zhang, S.; Li, Y.; Wu, C.; Zhang, W.; Zhang, H.; Xie, Z.; Zhou, S., Ultra-broadband random laser and white-light emissive carbon dots/crystal in-situ hybrids. *Small* **2022,** *18*, 2203152.

(8) Zhang, Y.; Hu, Y.; Lin, J.; Fan, Y.; Li, Y.; Lv, Y.; Liu, X., Excitation wavelength independence: Toward low-threshold amplified spontaneous emission from carbon nanodots. *ACS Appl. Mater. Interfaces* **2016,** *8*, 25454-25460.
